# Commentary: Is there any Influence of Variations in Context on Object-Affordance Effects in Schizophrenia? Perception of Property and Goals of Action

**DOI:** 10.3389/fpsyg.2016.01915

**Published:** 2016-12-08

**Authors:** Thomas J. Faulkenberry, Luca Tummolini

**Affiliations:** ^1^Department of Psychological Sciences, Tarleton State UniversityStephenville, TX, USA; ^2^Institute of Cognitive Sciences and Technologies, Italian National Research CouncilRome, Italy

**Keywords:** Bayes factors, null effects, significance testing, *p*-values, statistical power

In their recent article, Sevos et al. (2016) present data indicating that subjects with schizophrenia “have an impaired ability to experience an internal simulation of motor action potentialities when they perceived graspable objects” (p. 12). This lack of sensorimotor facilitation in patients with schizophrenia aligns broadly with other patient studies, and indicates that such individuals would require extensive use of higher cognitive processes even for the simplest routine activities in their daily life. A solid conclusion from this data would be certainly informative to understand the specific mechanisms behind schizophrenia.

The purpose of this commentary is to raise a point for further discussion. The claim that patients with schizophrenia lack this sensorimotor facilitation is based upon two non-significant effects reported in Experiments 1 and 2 of Sevos et al. (2016). This is problematic, though, since the traditional null-hypothesis testing approach does not allow one to “accept” a null hypothesis. This is because the *p*-value represents the probability of obtaining a sample statistic at least as large as that obtained from a given sample, assuming that the null hypothesis is true. If this *p*-value is small, we reject the null hypothesis on the grounds that an obtained sample statistic occurs with such low probability that the underlying null hypothesis should be considered implausible. If the *p*-value is not small, our only decision is to “fail to reject” the null hypothesis. It is important to note that this procedure only results in a decision to either reject or fail to reject; it does not provide any measure of evidence in favor of either the null or alternative hypothesis.

One common approach to help mitigate this problem is to report power. Mathematically, a test with sufficient power is less likely to produce a Type II error, and this allows one to feel somewhat assured that reported null effects are not simply false negatives. Though better than nothing, this approach still does not give any direct measure of evidence supporting an obtained null effect. However, recent methods based on Bayesian inference (Wagenmakers, 2007; Rouder et al., 2009) provide a relatively easy solution to this problem.

Though the specifics of Bayesian inference are beyond the scope of this short commentary (see Wagenmakers, 2007 for more details), the basic idea is that one computes a Bayes factor to index the preference for one model over another. The larger the Bayes factor, the more evidence in support of the model. One particular advantage to this approach is that it allows a researcher to directly assess evidence in support of the null hypothesis *H*_0_ over another hypothesis *H*_1_; such a Bayes factor would be denoted *BF*_01_. This Bayes factor represents the odds in favor of the null hypothesis over the alternative hypothesis after the data have been observed. Further, *BF*_01_ can be converted into a posterior probability, which is the probability that the null hypothesis *H*_0_ is true given data *D*.

To this end, we will describe how to compute *BF*_01_ and the posterior probabilities for the null effects reported in Experiments 1 and 2 of Sevos et al. (2016).

The first step in the computation is estimating the Bayes factor *BF*_01_. Following Wagenmakers (2007), Masson (2011) describes one approach to estimating *BF*_01_ that is based on the Bayesian Information Criterion, or BIC. *BF*_01_ is estimated as

(1) $$\begin{array}{r} BF_{01}\sim e^{\left(\Delta BIC/2\right)} \end{array}$$

where

(2) $$\begin{array}{r} \Delta BIC=n\ln\;\left(1-\eta_{p}^{2}\right)+\left(k_{1}-k_{0}\right)\ln\left(n\right). \end{array}$$

In Equation (2), *n* represents the number of subjects, $\eta_{p}^{2}$ is the standard effect size measure in an ANOVA which represents the proportion of variance accounted for by the independent variable, and *k*_1_ − *k*_0_ represents the difference in the number of free parameters between the two models being compared. Note that in the case of a comparison between a null and alternative hypothesis for a single two-level factor (i.e., prime, present vs. absent), *k*_1_ − *k*_0_ = 1. Finally, if we assume that the null and alternative hypothesis are equally likely before collecting data (that is, equal priors), the Bayes factor *B*_01_ can be converted into a posterior probability estimate via the equation:

(3) $$\begin{array}{r} p\left(H_{0}|D\right)=\frac{BF_{01}}{BF_{01}+1}. \end{array}$$

Now, let us compute Bayes factors for the reported null effects in Experiment 1 and 2 of Sevos et al. (2016). In Experiment 1, the authors reported that for the *n* = 18 patients with schizophrenia, the critical interaction of response and orientation did not differ as a function of name prime (present vs. absent), *F*_(1, 17)_ = 2.584, *p* = 0.126, $\eta_{p}^{2}=0.13$. Equation (2) yields

$$\begin{array}{l} \Delta BIC\;=n\ln\;\left(1-\eta_{p}^{2}\right)+\left(k_{1}-k_{0}\right)\;\ln\left(n\right)\\ \;=18\;\ln\left(1-0.13\right)+\left(1\right)\;\ln\left(18\right)\\ =0.384. \end{array}$$

Substituting this into Equation (1) then gives us the estimate

$$\begin{array}{l} BF_{01}\sim e^{\left(\Delta BIC/2\right)}\\ \;=e^{\left(0.384/2\right)}\\ \;=1.211. \end{array}$$

This means that, given the data, a null interaction is only 1.21 times more likely than a true interaction between response and orientation. According to Jeffreys (1961), Bayes factors falling between 1 and 3 are considered “anecdotal” evidence, whereas a Bayes factor between 3 and 10 represents “moderate” evidence, and a Bayes factor greater than 10 is considered “strong” evidence. As such, the evidence from Sevos et al. (2016) is anecdotal.

Additionally, we can use Equation (3) to compute the posterior probability of the null hypothesis:

$$\begin{array}{l} p\left(H_{0}|D\right)=\frac{BF_{01}}{BF_{01}+1}\\ \;=\frac{1.211}{1.211+1}\\ \;=0.55. \end{array}$$

According to Masson (2011), probability values falling between 0.50 and 0.75 are taken as weak evidence in favor of the null hypothesis. As the Bayes factor and posterior probability are directly related via Equation (3), they both tell the same story; that is, the null effect of name prime reported in Sevos et al. (2016) is not well supported.

A similar computation can be carried out for the effect of action prime in Experiment 2. Sevos et al. (2016) report that for a group of *n* = 18 patients with schizophrenia, the interaction between response and orientation did not differ as a function of action prime (congruent vs. incongruent), *F*_(1, 17)_ = 1.288, *p* = 0.272, $\eta_{p}^{2}=0.07$. Applying Equation (2) gives Δ*BIC* = 1.584, which implies (via Equation 1) that *BF*_01_ ~ 2.208, implying that the null interaction is only 2.21 times more likely than the true interaction. Equation 3 yields a posterior probability of *p*(*H*_0_|*D*) = 0.69. As with Experiment 1, evidence for this null effect is weak.

It is worth noting that this method is not the only approach to computing Bayes factors to assess null effects. The software package JASP (available as a free download from www.jasp-stats.org) contains a Summary Stats module that allows the user to compute Bayes factors from test statistics for a variety of common designs, including *t*-tests and linear regression. At present, the Summary Stats module does not have an option for ANOVA designs, in which case the method of Masson (2011) that we have presented provides a good solution. One should also note that Bayes factors and posterior probabilities can be computed directly from raw data using JASP or the BayesFactor package (Morey and Rouder, 2015) in R (R Core Team, 2016).

In summary, a Bayesian analysis of these two results indicates that at present, there is not much support for the null effects reported in Experiments 1 and 2. As such, any interpretations of these null effects should be met with caution. It is important to note that the points raised in this commentary are not meant to be unfairly critical of the results obtained by Sevos et al. (2016). On the contrary, the experiments are well-designed and informative, both in the context of embodied cognition as well as in the context of psychopathology. The purpose of this commentary was (1) to point out the issues that are present when trying to interpret nonsignificant results in the traditional null hypothesis statistical testing framework, and (2) to offer a quick example of how to use a Bayesian approach to quantify evidence for object-affordance effects and other action-specific influences on perception in the study of embodied cognition.

## Author contributions

TF wrote the draft of the manuscript and performed calculations, and LT provided revisions and clarifications.

### Conflict of interest statement

The authors declare that the research was conducted in the absence of any commercial or financial relationships that could be construed as a potential conflict of interest.
